# Round the Hospitals

**Published:** 1918-05-04

**Authors:** 


### ROUND THE HOSPITALS.

The Voting Papers in connection with the elections to the Council of the College of Nursing will be posted on Saturday, May 4. They must be received back at the office of the College, 6 Vere Street, Cavendish Square, London, W. 1, on or before Monday, May 20. Each nurse entitled to vote has a special responsibility to do her duty in support of the democratic basis of the College; and the surest way to fulfil it is to **complete the Voting Paper,** according to the directions, as soon as she receives it, **and to post it back to the College at the above address without delay.** We venture on these suggestions knowing by past experience how many votes are sometimes lost when Voting Papers are not immediately marked and posted. It is with great satisfaction we learn that the list of nominations is a thoroughly representative one, and that the number exceeds one hundred. The public and the profession will welcome this remarkable proof of the strength of the College. Friends and members of the College will rejoice and be glad with reason.

The efforts which Sir Arthur Stanley, to his lasting honour, is making throughout the country to enlighten the public on the need for an endowment of the Nursing Service are everywhere meeting with an enthusiastic response. In these matters nothing is wanted but a lead by a public man of position with a record of good work done, whose motives lie above suspicion and his assurance to the nation that the need exists. The rest follows as a matter of course. The growth and stability of the nursing profession are illustrated by the fact that the national tribute to nurses in this war will be associated with no one name. The endowment which started trained nursing in this country was originally a gift to Florence Nightingale. The endowment which made it possible to train district nurses in large numbers for service among the poor was a personal gift to Queen Victoria. In this instance the public are subscribing in full consciousness that their gifts will be used for the regeneration of the Nursing Service, for the promulgation of wise reforms, for the removal of that blot on the profession, the disabled nurse without friends or means. Sir Arthur Stanley was on very sure ground in his contention at Leicester that now is the psychological moment for a united effort by nurses and for nurses. Those at home must see to it that the ground is prepared in anticipation of the demobilisation which will be accomplished when peace is declared. Without this united effort, without the establishment of a representative Nursing Council, the future would be dark indeed, for the dissolution of military regulations and the return to civil occupations of from 20,000 to 30,000 nurses might land the nursing profession in a state of confusion little short of chaos.

Princess Mary, who has just come of age, is following in the steps of her mother, her grandmother, and great-grandmother in taking an absorbing interest in nursing. She is Commandant of a Voluntary Aid Detachment at Buckingham Palace, numbering twenty-five members, to whom Sir James Cantlie lectured on first-aid and nursing, and since last July she has passed her two examinations, oral, written and practical, and now holds certificates in first-aid and nursing. She spends three mornings a week at Devonshire House as a V.A.D. helper, assisting on the administrative side and gaining much practical insight into the management and organisation of that and kindred philanthropic undertakings. Wherever her future lot is cast, these regular labours, with the accompanying mastery of detail, will stand her in good stead. It makes all the difference to a charity, an institution, or a society whether the President is merely a figure-head or has a competent grasp of the problems which have to be faced, and since Princess Mary will, in all probability, be called to much responsible work for others, it is the happiest of auguries that she is grounding herself from the beginning in these matters, and is gaining inside knowledge of conditions which affect both nurses and patients.

All Army nurses receive a fixed messing allowance from their respective Governments, but the manner in which this allowance is spent differs widely in practice, and the subject of messing is exciting a good deal of attention at the present moment. The British system is for the catering to be done either by a sister or by a V.A.D. worker, appointed by the matron and subject to her orders. The Australian system provides for the issue of cooked rations wherever possible, and these are served from the same kitchen which serves the rations for the officers. The mess allowance is 2s. 6d. a day as a minimum, subject to increase where necessary. There is a Mess Committee among the sisters, which elects one of its own members as president. It meets in the evening as required, and complaints or petitions can be considered. In the New Zealand Nursing Service 3s. 6d. a day is allowed for messing. There is a Mess Committee on which the matron and sisters sit, but the actual catering is managed by someone put in charge by the matron, who may be either one of the sisters or a lay woman should such be available. It seems on the face of it more reasonable for the catering to be undertaken in war-time by a woman who is not a trained nurse, for there are undoubtedly periods in every war hospital when the whole time of every skilled nurse is needed for nursing duties. Either the catering or the nursing must go to the wall at such times, if the housekeeper is a nursing sister. But the principle of a Mess Committee, however variously it may be worked, is thoroughly sound. Where supplies are short and hardships have to be borne, it makes all the difference if inside information about catering difficulties is attainable. It is an instinct in women to demand some share, if only indirect, in managing their own commissariat, and where this privilege is denied, as in institutions and hotels, there the voice of the grumbler is seldom long silent.

THE vexed question of smoking among nurses is receiving considerable attention from the nurses who reach our shores from distant parts of the world. The extent to which Englishwomen, including English nurses, have succumbed to the cigarette habit stirs our new comrades with astonishment; shall we say disgust? One eminent Australian nurse has declared that Englishwomen consume more tobacco in a day than the inmates of an Egyptian hareem. Among Australian and New Zealand nurses the habit was unknown before they came to this country. It has few redeeming features. Its supposed refreshing effects on the nerves are the offspring of suggestion and are grossly exaggerated by its votaries, while when carried to excess its deteriorating effects in the same direction are notorious. From the nursing point of view there is, we imagine, nothing to be said for the cigarette. It imparts an air of "commonness" to the wearer of uniform wherever it is exhibited. It taints the breath unpleasantly. It stains the finger-tips. It runs away with a vast amount of money. Women's smoking is purely imitative and as a habit is out of keeping with the spirit of nursing.

THE section on the nursing department of St. George's Hospital contained in the annual report for 1917 presents several features of interest. It will be remembered that Miss Cooper, who held the office of matron for about two years, resigned last year on her marriage, and that her place was filled by Miss Babtie, whose close connection with the hospital made the transition an easy one. The nursing staff consists of twenty-five sisters and 100 nurses and probationers. Twenty-two nurses completed their training during the year, of whom fourteen have joined the Military or Naval Reserves and the British Red Cross. Of the rest, two are taking maternity training, one is doing private work, two have been made sisters at St. George's, and three have gone home. This account throws some light on the proportion of newly trained women who can be counted on for Army service. Forty-three new probationers have entered for training, this being apparently much above the average for the year, and five war probationers have been admitted for a short course. The normal number of new probationers in a hospital with a four years' course is one-fourth of the nursing staff, exclusive of sisters, so that, if all went automatically, twenty-five nurses would finish their term and would be replaced by twenty-five others. But losses from illness or family affairs easily upset this nicely balanced system. That the St. George's nurses have had their share of war work is shown by the fact that 514 sick and wounded soldiers have been nursed at the hospital during the year.

FEW matrons have been privileged to rule over a fairer domain than that offered by Fulham Palace, recently converted into a military hospital, to which Mrs. M. Fox-Symons has been appointed matron. The Old Court is a fine specimen of architecture, and, like all the spacious Tudor buildings which have fallen into the hands of the Red Cross, is finely adapted for the nursing of the wounded. The Bishop of London has never been better inspired than in making this surrender. His example is likely to be followed, and already it is announced that the Bishop of Norwich has given up his palace and grounds to form a V.A.D. Hospital with forty beds. This palace, like Fulham, is of high antiquity, though much modernised, and there is a beautiful Early Decorated ruin in the garden; altogether a peaceful and unique setting for the cure of the wounded.

IT is not often in the present day that a matron during her term of office can have the gratification of beholding her institution raised from a public scandal into a model hospital. This, however, has been the experience of Miss Braidwood, matron of the Myland Infectious Hospital, Colchester. The Borough and Port Health Committee have recently recorded on the minutes a letter from Dr. A. Nolan Fell, expressing his admiration for the splendid work going on at the Borough Hospital under Miss Braidwood and Sisters Hayes and Dale, "in whom the Committee possess officials of the highest capacity." If this were all, it would be much. But we have a copy of a letter written by a patient in 1901, after a stay of some weeks in this very hospital. Grateful for his recovery, he writes in a tone of studied moderation to recount his experiences prior to the arrival of Miss Braidwood and the instalment of a trained staff. No bath-room at all, no water accessible from the wards, no foot-pan, even; earth closets leading out of the wards emptied only every few days; rotten flooring; ceiling low enough to touch with the hand; rats and mice abounding; rain coming through from above and draughts from beneath. An epidemic of diphtheria was in progress, and the staff for fourteen patients consisted of one nurse and one girl of seventeen, who lay down in the wards at night to snatch a little sleep. The sleeping quarters of the staff led off the same staircase and landing as that used by diphtheria and scarlet-fever patients. Such was the state of affairs when Miss Braidwood came on the scene. With characteristic modesty she remarks there have been many difficulties and much hard work during these seventeen years, "but I am fortunate in my staff." It is a curious thing that matrons who accomplish great things invariably tell us they are "fortunate in their staff."

IN the reference to the Kensington Infirmary last week the words "nursing staff" should have been "probationary nursing staff." Including sisters, the total staff in normal times now numbers close on 100, and only lack of proper accommodation prevents its increase. The forced inaction prescribed on managers of institutions like the Kensington Infirmary, which present conspicuous deficiencies in respect of the housing of the nursing staff, ought not, in our judgment, to extend to brain work. There will be enormous competition for building material and labour when the embargo on building is removed, and those will come off best who have already made out their plans and know exactly what they want to have done. It will be no more easy then than now to find space for a new Nurses' Home, and the interval could be wisely utilised in the solution of the problem how best to redeem the infirmary from a stigma which is retarding its development as a municipal hospital.

Sixty-four out of every hundred children at Leeds have been found on medical examination to be deficient in one way or another. This startling announcement was made by Miss Curtis lately in speaking at the annual meeting of the Bradford Maternity Care Committee. Both at Leeds and Bradford strenuous efforts are being made to reduce the infantile death-rate and lessen the amount of disease among children. Leeds has already nine centres for maternity work, and is opening a tenth, for it is found that the secret of success in these undertakings is to bring them almost to the door of the mothers. Bradford is still hampered by want of money; but many different ways of helping the mothers are being tried, and at three centres communal dinners are served. The importance of keeping fresh in theory and practice is fully realised up North. The prevalence of chronic ill-health among children throughout the kingdom can only be reduced by avoiding stereotyped methods, and by personally influencing the mothers one by one to adopt a higher standard of family life.

It is not long since we commented on the efficient protection afforded to the V.A.D. uniform by the county badge and numeral. The ease with which infringements of the system can be detected is illustrated by the case of the daughter of an officer, who has been fined £20 at Southampton for wearing the uniform of a Red Cross nurse without being entitled to do so. Had she confined herself to posing as a fully-trained nurse by imitating the uniform of any civilian nursing institution, no remedy could have been found and no penalty inflicted.

